# MIGS: A Modular Edge Gateway with Instance-Based Isolation for Heterogeneous Industrial IoT Interoperability

**DOI:** 10.3390/s26010314

**Published:** 2026-01-03

**Authors:** Yan Ai, Yuesheng Zhu, Yao Jiang, Yuanzhao Deng

**Affiliations:** 1 Faculty of Data Science, City University of Macau, Macau 999078, China; d23092110311@cityu.edu.mo (Y.A.); d23092110029@cityu.edu.mo (Y.J.); d23092110010@cityu.edu.mo (Y.D.); 2 Faculty of Artificial Intelligence, Guangdong Industry Polytechnic University, Guangzhou 510300, China; 3 School of Information Technology, Peking University, Shenzhen 518055, China

**Keywords:** IoT gateway, multi-protocol interoperability, edge computing, system architecture, industrial IoT (IIoT), heterogeneous networks

## Abstract

The exponential proliferation of the Internet of Things (IoT) has catalyzed a paradigm shift in industrial automation and smart city infrastructure. However, this rapid expansion has engendered significant heterogeneity in communication protocols, creating critical barriers to seamless data integration and interoperability. Conventional gateway solutions frequently exhibit limited flexibility in supporting diverse protocol stacks simultaneously and often lack granular user controllability. To mitigate these deficiencies, this paper proposes a novel, modular IoT gateway architecture, designated as MIGS (Modular IoT Gateway System). The proposed architecture comprises four distinct components: a Management Component, a Southbound Component, a Northbound Component, and a Cache Component. Specifically, the Southbound Component employs instance-based isolation and independent task threading to manage heterogeneous field devices utilizing protocols such as Modbus, MQTT, and OPC UA. The Northbound Component facilitates reliable bidirectional data transmission with cloud platforms. A dedicated Cache Component is integrated to decouple data acquisition from transmission, ensuring data integrity during network latency. Furthermore, a web-based Control Service Module affords comprehensive runtime management. We explicate the data transmission methodology and formulate a theoretical latency model to quantify the impact of the Python Global Interpreter Lock (GIL) and serialization overhead. Functional validation and theoretical analysis confirm the system’s efficacy in concurrent multi-protocol communication, robust data forwarding, and operational flexibility. The MIGS framework significantly enhances interoperability within heterogeneous IoT environments, offering a scalable solution for next-generation industrial applications.

## 1. Introduction

The Internet of Things (IoT) has emerged as a transformative force across diverse sectors, ranging from Industry 4.0 and smart manufacturing to healthcare and urban infrastructure [1,2]. By facilitating the interconnection of physical entities and enabling ubiquitous data exchange, IoT ecosystems promise unprecedented levels of operational efficiency and Near real-time monitoring. Recent market analyses project the global industrial IoT gateway market to expand significantly, driven by the escalating demand for connectivity in legacy industrial environments [3]. However, this expansion is characterized by a fragmented ecosystem populated by a plethora of communication protocols. In industrial settings, legacy fieldbus protocols such as Modbus, Profinet, and EtherCAT remain prevalent, while modern machine-to-machine (M2M) communications increasingly rely on lightweight protocols like MQTT and interoperability standards like OPC UA [4,5]. This protocol heterogeneity constitutes a substantial impediment to the realization of unified IoT platforms, creating “data silos” that hinder scalable deployment [6].

IoT gateways function as pivotal intermediaries within this landscape, bridging the operational technology (OT) and information technology (IT) divide [7]. Their primary mandate involves protocol translation, data aggregation, and secure transmission to cloud-based analytics platforms. Despite the availability of commercial gateway solutions, significant challenges persist. Many existing gateways utilize monolithic architectures with hard-coded protocol logic, restricting their adaptability to evolving device requirements [8]. Furthermore, commercial solutions often prioritize vendor-specific ecosystems, leading to interoperability lock-in and inflated integration costs. A critical gap also exists in management flexibility; many gateways rely on static configuration files or command-line interfaces, which are ill-suited for dynamic industrial environments requiring frequent reconfiguration [9].

While concepts such as modularity and caching have been explored in high-end NFV/SDN-based gateways [10] and BIM-oriented platforms [11], these solutions often require substantial computational resources (e.g., virtualization overhead) or lack the agility for runtime reconfiguration without system restarts. MIGS differentiates itself by offering a lightweight, Python-based instance isolation mechanism that operates effectively on constrained edge hardware (Raspberry Pi class) while providing granular, zero-downtime control over individual device connections—a feature often absent in static open-source baselines.

To mitigate these deficiencies, this paper proposes MIGS (Modular IoT Gateway System), a novel, lightweight, and software-defined gateway architecture. MIGS is designed to provide a robust, flexible, and user-friendly solution for heterogeneous device integration.

The specific contributions of this paper are as follows:

- Modular Architecture Design: We propose a four-component architecture (Management, Southbound, Northbound, Cache) that enhances system scalability and maintainability.
- Instance-Based Connection Management: We introduce a dynamic connection mechanism where each device interface is encapsulated in an independent task thread, preventing single-point failures and ensuring isolation between heterogeneous protocols.
- Decoupled Data Transmission: We describe a robust data handling methodology utilizing an intermediate cache layer to buffer data, ensuring reliability against network volatility.
- Comprehensive Validation: We provide an empirical evaluation of the system’s latency, throughput, and resource utilization under multi-protocol conditions, demonstrating its suitability for industrial applications.
- Theoretical Performance Modeling: We provide a mathematical model for end-to-end system latency, analyzing the constraints of the GIL and serialization processes to identify theoretical throughput bottlenecks.

The remainder of this paper is organized as follows: Section 2 reviews the state of the art in IoT gateway architectures, focusing on interoperability and edge computing trends. Section 3 details the proposed MIGS architecture and its core components. Section 4 describes the hardware and software implementation specifics. Section 5 explicates the data transmission methodology and theoretical latency model. Section 6 presents a comprehensive performance evaluation, including latency, throughput, and scalability analysis. Finally, Section 7 concludes the study and outlines future research directions.

## 2. State of the Art

### 2.1. IoT Gateway Architectures and Interoperability Challenges

The challenge of heterogeneity in IoT has been extensively studied in recent years, with interoperability remaining a primary barrier to widespread adoption [12]. Traditional gateways often function as simple packet forwarders, but the trend is shifting towards “smart” gateways capable of edge processing and intelligent protocol translation [13].

Early gateway designs were predominantly monolithic, where the application logic and protocol drivers were tightly coupled. This rigidity made it difficult to add support for new protocols without significant system re-engineering [14]. Zhu et al. [15] emphasized the role of gateways in bridging wireless sensor networks, yet their approach lacked the modularity required for modern multi-protocol environments.

Recent surveys from 2023 and 2024 highlight that while hardware capabilities have improved, software interoperability remains fragmented [16,17]. The concept of "semantic interoperability" has gained traction, with researchers proposing ontology-based gateways to translate raw data into meaningful information contexts [18]. However, these semantic approaches often incur high computational overhead, making them less suitable for resource-constrained edge devices compared to syntactic translation methods utilized in lightweight gateways [19].

Recent studies have proposed distributed IoT gateways leveraging NFV and SDN for disaster management [10]. While highly scalable, these architectures introduce complexity unsuitable for standalone industrial cells. Similarly, semantic gateways for building information modeling [11] focus on data interoperability but often neglect the deterministic timing requirements of real-time fieldbus acquisition. MIGS positions itself as a middle-ground solution: lightweight enough for embedded deployment yet robust enough for multi-protocol concurrency.

### 2.2. Protocol Integration in Industrial Environments

The coexistence of legacy and modern protocols is a defining feature of current IIoT deployments. Modbus remains the de facto standard for low-level device communication due to its simplicity and ubiquity, despite its lack of security and metadata capabilities [20]. Conversely, OPC UA is gaining traction for its robust security and rich information modeling, serving as a key enabler for Industry 4.0 [21].

For northbound communication, MQTT has established itself as the standard due to its lightweight publish/subscribe model, which is ideal for bandwidth-constrained networks [22]. Integrating these diverse protocols requires sophisticated handling. A 2023 study by Hameed et al. [23] identified “heterogeneity in communication” as a primary challenge, noting that translating between synchronous (e.g., Modbus polling) and asynchronous (e.g., MQTT publish) models often leads to data loss or latency issues if not managed correctly.Recent implementations have also demonstrated the efficacy of bridging legacy Modbus networks with MQTT for cloud integration [24].

Commercial platforms like AWS IoT Greengrass [25] and Azure IoT Edge [26] have popularized containerized edge computing, allowing developers to deploy cloud logic to the edge. However, these platforms can be resource-intensive and often require specific hardware dependencies or vendor lock-in. In contrast, open-source initiatives often struggle with the complexity of industrial protocols like OPC UA or lack user-friendly management interfaces [27].

### 2.3. Edge Computing and Latency Optimization

Edge computing has emerged as a critical paradigm to address the latency and bandwidth limitations of cloud-centric IoT architectures [28]. By processing data closer to the source, gateways can significantly reduce the volume of data transmitted to the cloud and enable real-time decision-making [29]. Recent research has focused on optimizing task offloading and resource allocation at the edge. For instance, reinforcement learning-based approaches have been proposed to dynamically allocate gateway resources based on network conditions [30]. However, for many industrial applications, deterministic performance is more critical than dynamic optimization. Our work focuses on a deterministic, instance-based threading model to ensure predictable latency and isolation for critical control tasks, addressing a gap in lightweight, deterministic gateway designs [31].

## 3. System Architecture

**Figure 1:**
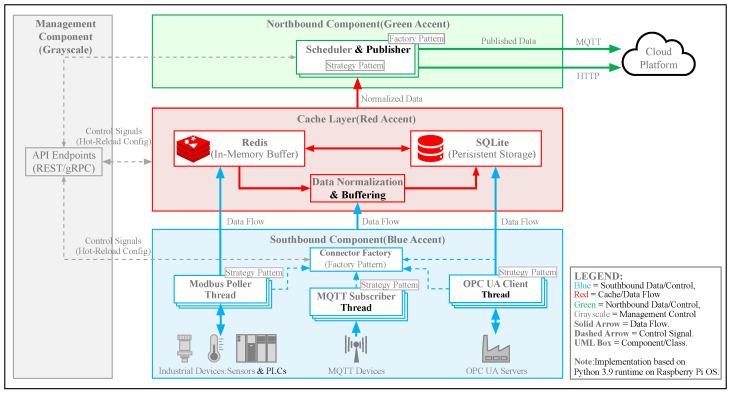
Overall architecture of the proposed IoT gateway system (MIGS).

The overall architecture of the proposed MIGS is constructed upon principles of modularity, loose coupling, and high cohesion. As illustrated in Figure 1, the system is stratified into four interconnected components: the Management Component, Southbound Component, Northbound Component, and Cache Component. This separation of concerns facilitates independent development, testing, and scaling of each module.

The core responsibilities of each component are summarized in Table 1.

### 3.1. Management Component

This component functions as the orchestration engine of the gateway, responsible for configuration management and user interaction.

- Control Service Module: Hosting a RESTful web server (Port 80), this module provides the Human-Machine Interface (HMI) for system administration. It interprets user commands (e.g., device provisioning, protocol configuration) and dispatches them to the core logic via internal IPC mechanisms.
- IoT Service Module: This module encapsulates internal service providers, including a local MQTT broker (Port 1883) and an OPC UA server (Ports 4880/4881). This design allows the gateway to function as a local data aggregator and server, enabling local control loops even when external cloud connectivity is severed.
- Database Module: Utilizing SQLite for configuration persistence, this module stores device profiles, register maps, and network settings. The use of a relational database ensures data integrity and simplifies complex queries for device management.
- OS Management Module: This module encapsulates operating system-level operations, specifically network configuration (e.g., 4G/Ethernet monitoring) and system performance monitoring. It provides real-time metrics such as CPU usage, memory footprint, disk space, and system load, facilitating proactive health monitoring and fault diagnosis.

### 3.2. Southbound Component

The Southbound Component is engineered to abstract the complexity of physical device interactions and protocol diversity. Its design leverages robust software engineering patterns to ensure extensibility and maintainability.

**Figure 2:**
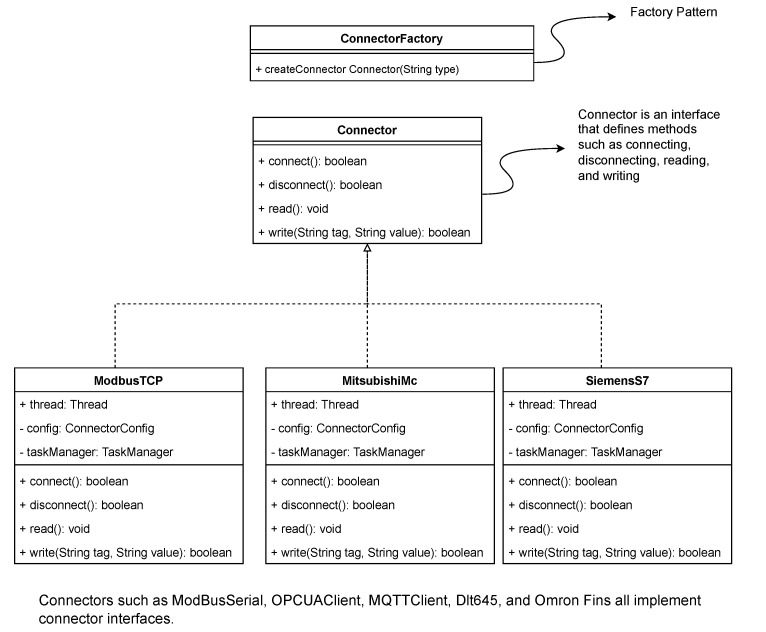
UML Class Diagram of the Connector Factory and Strategy Pattern. The Connector interface defines the contract, while concrete classes (e.g., ModbusConnector) implement specific protocols.

**Figure 3:**
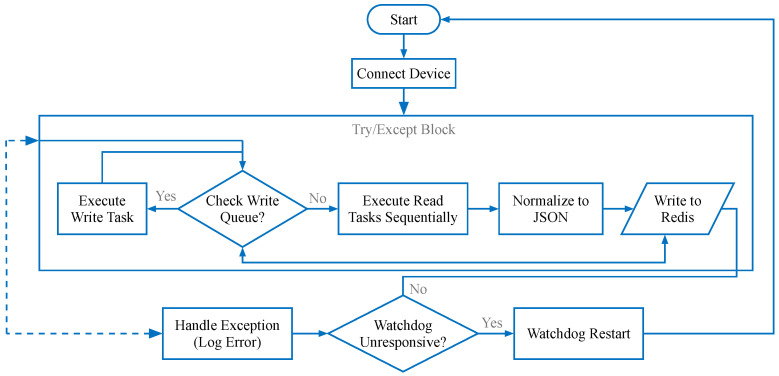
Task Manager Flowchart. The internal loop of a connector thread, showing the prioritization of write commands over read tasks.

**Figure 4:**
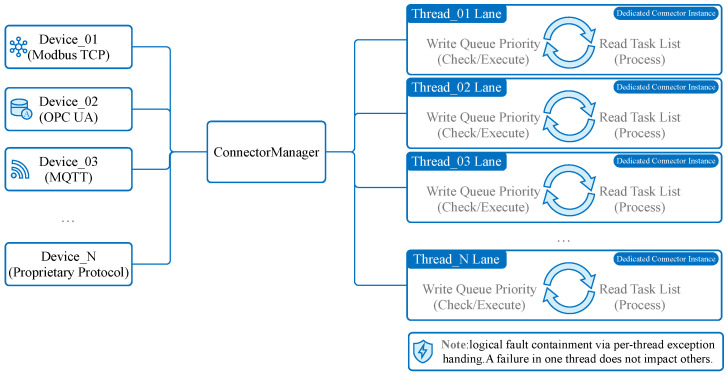
Connection Relationship Diagram. Each device connection corresponds to a unique Connector instance running in a dedicated thread, managed by the ConnectorManager.

- Connector Manager: This sub-module oversees the lifecycle of all active connectors. It is responsible for instantiation, starting, stopping, and error recovery of connector instances.
- Connectors (Strategy & Factory Pattern): To support a wide array of protocols (Modbus, Siemens S7, etc.) while adhering to the Open/Closed Principle (OCP), we employ a combination of the Strategy Pattern and Factory Pattern. A unified Connector interface defines standard methods (connect, disconnect, read, write). Specific protocol implementations (e.g., ModbusConnector, OpcUaConnector) implement this interface. At runtime, the Connector Manager uses a Factory to dynamically instantiate the correct strategy based on the configuration. This design allows new protocols to be added by simply creating a new class without modifying existing code. As illustrated in Figure 2.
- Task Manager: Each connector instance maintains an internal Task Manager that orchestrates specific data acquisition tasks. As illustrated in Figure 3. A “Task” encapsulates the atomic details of a read operation, such as register address, length, data type, and scaling formulas.
- Instance-Based Isolation: A core innovation of MIGS is its instance-based threading model. For every connected device, a dedicated, isolated thread is spawned.As illustrated in Figure 4. This “one-thread-per-device” architecture ensures that a timeout, blocking operation, or crash in one device driver does not affect the execution of other device tasks.It is important to note that this provides logical fault containment rather than strict OS-level process isolation. While threads share the same memory space and Global Interpreter Lock (GIL), our exception handling wrapper (described in Section 4.4) ensures that a logical error (e.g., protocol parsing failure, device timeout) in one thread is caught and handled locally, preventing it from crashing the main application loop. For catastrophic failures (e.g., segfaults in C-extensions), we recommend external process supervision (e.g., systemd).
  **Note:** While MQTT is typically a Northbound protocol, its inclusion in the Southbound component facilitates integration with other smart sensors or sub-gateways that publish data actively. In this context, the Southbound MQTT Connector acts as a subscriber.

### 3.3. Cache Component

To address the impedance mismatch between high-frequency polling (Southbound) and event-driven transmission (Northbound), a Cache Component is introduced. Implemented using Redis, it serves as a high-speed, in-memory key-value store.

- Decoupling: Southbound tasks write normalized data to the cache immediately upon acquisition. The Northbound Component reads from the cache at its own scheduled intervals.
- Data Normalization: Before storage, raw data from different protocols is normalized into a unified JSON structure (see Section 5.2), ensuring that the Northbound component is agnostic to the source protocol.
- Buffering: The cache provides a temporary buffer, preventing data loss during brief network outages or high-load spikes.

### 3.4. Northbound Component

This component handles upstream connectivity and data egress.

**Figure 5:**
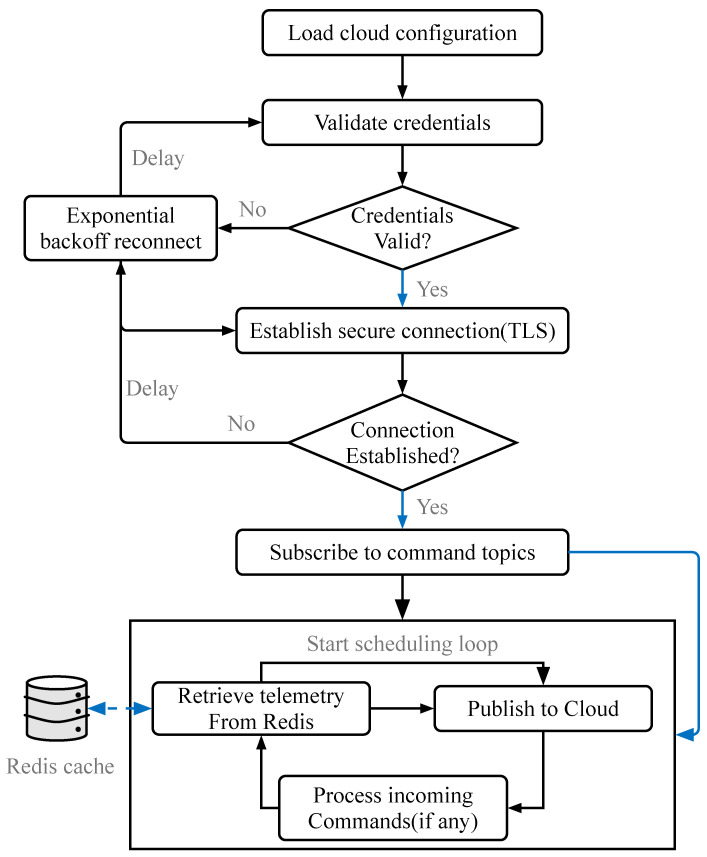
Northbound Cloud Manager Initialization. The flow for reading cloud configuration and establishing the uplink connection.

**Figure 6:**
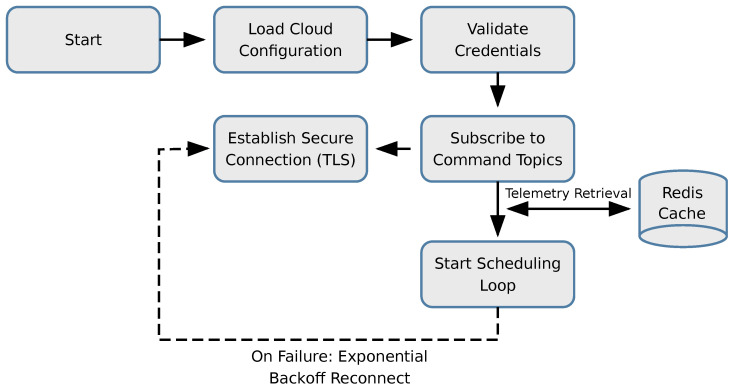
Northbound Scheduling Process. The cyclic process of polling the cache, serializing data, and publishing to the cloud.

- Cloud Platform Manager: This module manages the lifecycle of cloud connections. It supports dynamic configuration of multiple cloud platforms (e.g., AWS IoT, ThingsBoard, Aliyun). As illustrated in Figure 5.
- Scheduling Module: This module implements a configurable polling loop that retrieves fresh data from the Cache Component, As illustrated in Figure 6. It encapsulates the data into standard JSON payloads and transmits them via MQTT or HTTP/S. Additionally, it subscribes to downstream command topics, parsing cloud-originated control instructions and routing them to the appropriate Southbound device instance for execution.

### 3.5. Security Architecture

Security is paramount in IIoT deployments. MIGS incorporates a multi-layered security strategy:

- Transport Security: All Northbound communications are encrypted using TLS 1.2/1.3 to prevent eavesdropping and tampering.
- Access Control: The web interface is protected by role-based access control (RBAC) and token-based authentication.
- Isolation: The process isolation between Southbound drivers prevents a compromised or malfunctioning device driver from crashing the entire system kernel.

## 4. Implementation Details

The prototype of MIGS was implemented to validate the architectural design and evaluate its performance in real-world scenarios.

### 4.1. Hardware Platform Specification

The system is deployed on a Raspberry Pi 4 Model B, selected for its favorable performance-to-cost ratio and extensive I/O capabilities, making it a representative edge computing node.

- SoC: Broadcom BCM2711, Quad-core Cortex-A72 (ARM v8) @ 1.5 GHz.
- Memory: 4 GB LPDDR4 SDRAM.
- Connectivity: Gigabit Ethernet, 2.4/5.0 GHz 802.11ac Wi-Fi, Bluetooth 5.0.
- Storage: 32 GB Class 10 MicroSD for OS and application binaries; external SSD support via USB 3.0 for local data logging.

Rationalefor Platform Selection: While industrial microcontrollers (e.g., STM32L4 series) running Real-Time Operating Systems (FreeRTOS) offer superior deterministic performance and lower power consumption, they often lack the resources to support high-level dynamic languages and containerization (Docker) central to the MIGS architecture. The Raspberry Pi 4 serves as a representative “High-Level OS” gateway, prioritizing development flexibility, rich Northbound connectivity (SSL/TLS, JSON), and ease of reconfiguration over hard real-time execution. For industrial hardening, the software stack is designed to be portable to industrial-grade Linux gateways (e.g., Siemens IOT2050, Moxa UC-8100).

### 4.2. Software Stack and Rationale

- Operating System: Raspberry Pi OS (Debian 11 Bullseye, 64-bit) provides a stable, Linux-based foundation with widespread driver support.
- Programming Language: Python 3.9 is utilized for the core application logic. While C++ offers higher raw performance, Python was chosen for its rapid development cycle, rich ecosystem of IoT libraries (e.g., pymodbus, paho-mqtt), and ease of maintenance. Performance-critical sections, such as the underlying protocol drivers, often rely on C-based extensions, mitigating the interpretation overhead.
- Data Store:
  − SQLite 3: Used for persistent storage of configuration data. Its serverless, file-based nature makes it ideal for embedded devices where a full-fledged SQL server would be overkill.
  − Redis 6.2: Employed for the Cache Component. Its in-memory nature ensures microsecond-level read/write latency, which is crucial for high-frequency data buffering.
- Containerization: Docker is employed to containerize the application services. This ensures consistent deployment environments across different hardware and simplifies the update process via image replacement.

### 4.3. Protocol Library Integration

- Modbus: The pymodbus library is used for both TCP and RTU communications. We implemented a custom wrapper to handle automatic reconnection with exponential backoff strategies and CRC error checking.
- MQTT: The Eclipse Paho MQTT Python client is integrated, supporting QoS levels 0, 1, and 2, as well as Last Will and Testament (LWT) for connection status monitoring.
- OPC UA: The FreeOpcUa (client) and open62541 (server wrapper) libraries are used. Support includes basic username/password authentication and Basic256Sha256 security policies.

### 4.4. Error Handling and Resilience

To ensure industrial-grade reliability, robust error handling mechanisms were implemented:

- Watchdog Timer: A software watchdog monitors the status of all critical threads. If a thread becomes unresponsive, it is automatically terminated and restarted.
- Exception Isolation: Each device thread runs within a try-except block to catch unhandled exceptions, logging them to a rotating log file for post-mortem analysis without affecting the main process.

## 5. Data Transmission Method

The operational logic of MIGS follows a deterministic state machine model, ensuring predictable behavior during system startup, runtime, and shutdown.

### 5.1. System Initialization Sequence

**Figure 7:**
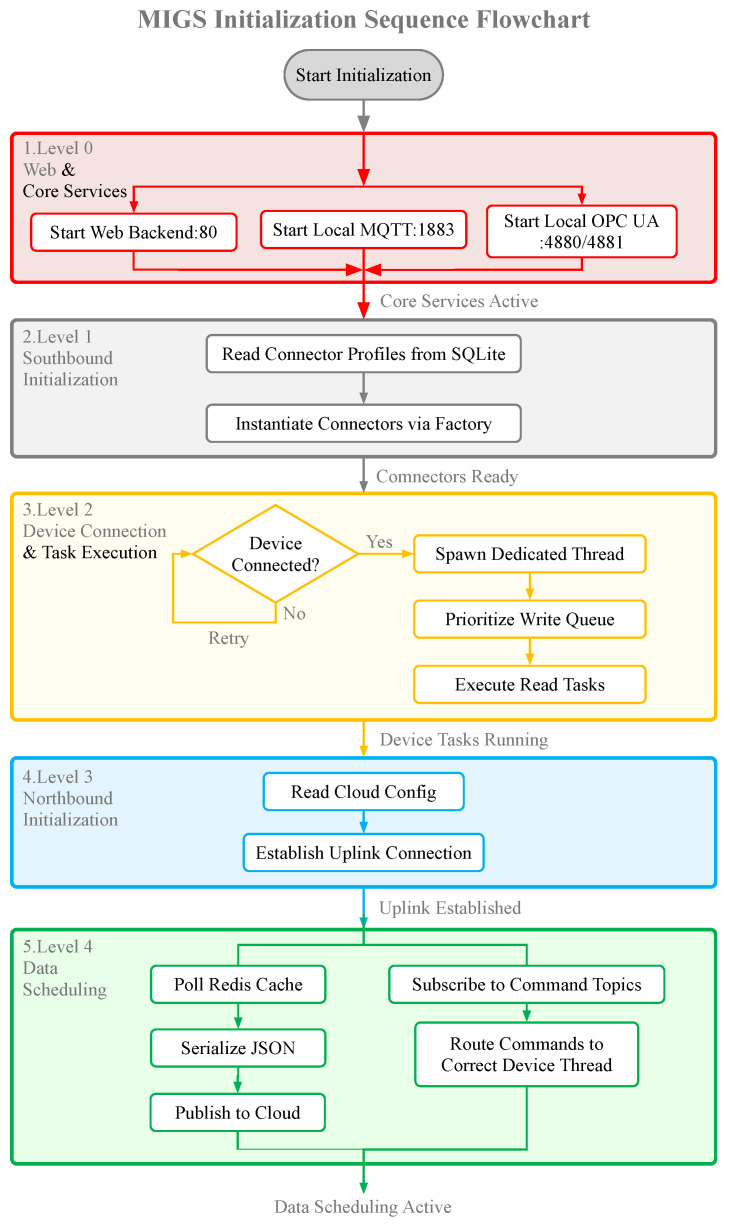
Initialization Sequence/Data Transmission Flow.

Upon system boot, As illustrated in Figure 7, the initialization follows a strict dependency order:

- Level 0 (Core): The OS Module initializes system resources and mounts the file system.
- Level 1 (Persistence): The Database Module loads and verifies the integrity of the configuration files.
- Level 2 (Services): The Management Component starts the web server and internal MQTT/OPC UA brokers.
- Level 3 (Southbound): The Task Management Module queries the database for active device profiles. For each enabled profile, it instantiates the corresponding protocol object and spawns a dedicated data acquisition thread.
- Level 4 (Northbound): The Northbound Component establishes a secure socket connection to the configured cloud endpoint and begins the scheduling loop.

### 5.2. Data Normalization Strategy

A key function of MIGS is converting heterogeneous raw data into a unified format. We employ a flat JSON schema for internal data representation:



{
  "timestamp": 1678892345,
  "device_id": "meter_01",
  "protocol": "modbus_tcp",
  "data": {
    "voltage_a": 220.5,
    "current_a": 5.2,
    "power_active": 1146.6
  },
  "status": "ok"
}
        


This schema allows the Northbound component to serialize and transmit data without needing to understand the specific details of the source protocol (e.g., Modbus register addresses).

### 5.3. Runtime Control Operations

The system supports dynamic reconfiguration through the Control Service Module, allowing operators to manage the gateway without downtime. The logic for these operations ensures data integrity:

- Start: The Connector Manager establishes the connection. If successful, it launches the read/write thread.
- Pause: The system signals the thread to terminate gracefully and then closes the physical connection, retaining the configuration in memory.
- Add: The system first validates the user input (e.g., IP address format, register range). If valid, the configuration is written to the database, and the Connector Manager is notified to instantiate the new connector.
- Delete: The system performs a safety check to see if the connector is currently active. If yes, it first triggers the "Pause" logic to stop the thread and release resources. Only after the thread is confirmed stopped is the configuration removed from the database.
- Update: Similar to “Add”, the system validates the new parameters. If valid, it updates the database. If the connector was running, it triggers a “hot reload” (Pause -> Re-instantiate -> Start) to apply changes immediately.

### 5.4. Theoretical Modeling of System Latency

**Figure 8:**
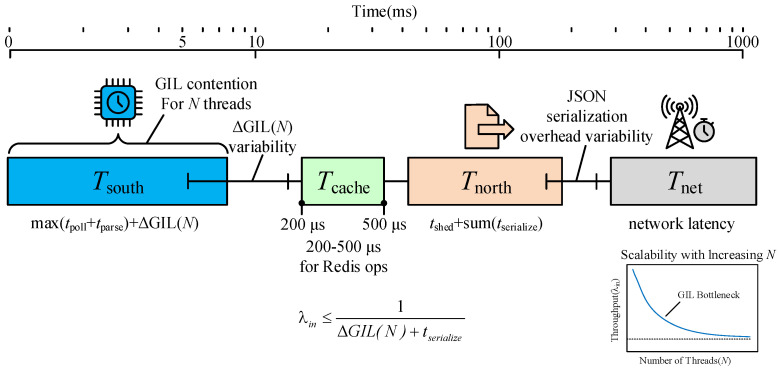
Theoretical Latency Model Pipeline.

To rigorously analyze the temporal behavior of MIGS, we formulate a theoretical latency model based on its decoupled “Southbound–Cache–Northbound” architecture. As illustrated in Figure 8. Unlike monolithic gateways where data flows synchronously from source to sink, MIGS introduces a buffering stage. The total end-to-end latency, denoted as $T_{\mathrm{total}}$, can be modeled as the sum of four distinct temporal components:

(1) $$T_{\mathrm{total}}=T_{\mathrm{south}}+T_{\mathrm{cache}}+T_{\mathrm{north}}+T_{\mathrm{net}}$$

where:

- $T_{\mathrm{south}}$ represents the time required to acquire data from field devices.
- $T_{\mathrm{cache}}$ is the latency introduced by the intermediate Redis storage.
- $T_{\mathrm{north}}$ denotes the processing time for the Northbound component to retrieve and serialize data.
- $T_{\mathrm{net}}$ is the network transmission delay to the cloud.

#### 5.4.1. Parallel Acquisition Latency ($T_{\mathrm{south}}$)

A critical advantage of our instance-based isolation is parallel execution. For a set of *N* heterogeneous devices, the acquisition time is determined by the slowest device thread rather than the sum of all polling times. However, due to the Global Interpreter Lock (GIL) in CPython, true parallelism is constrained. The effective southbound latency is modeled as:

(2) $$T_{\mathrm{south}}=\max_{i=1}^{N}\left(t_{\mathrm{poll}}^{\left(i\right)}+t_{\mathrm{parse}}^{\left(i\right)}\right)+\Delta_{\mathrm{GIL}}\left(N\right)$$

Here, $t_{\mathrm{poll}}^{\left(i\right)}$ is the I/O blocking time for device *i* (e.g., waiting for a Modbus response), and $t_{\mathrm{parse}}^{\left(i\right)}$ is the CPU time for parsing the response. The term $\Delta_{\mathrm{GIL}}\left(N\right)$ represents the non-deterministic overhead introduced by thread context switching and GIL contention, which grows non-linearly as *N* increases.

#### 5.4.2. Serialization and Scheduling Latency ($T_{\mathrm{north}}$)

The Northbound component operates on a fixed schedule. Its latency is primarily CPU-bound, dominated by the JSON serialization of normalized data:

(3) $$T_{\mathrm{north}}=t_{\mathrm{sched}}+\sum_{k=1}^{M}t_{\mathrm{serialize}}^{\left(k\right)}$$

where $t_{\mathrm{sched}}$ is the scheduler wake-up jitter (typically negligible in low-load scenarios but significant under heavy load) and *M* is the number of data points to be transmitted in the current batch.

#### 5.4.3. Throughput Bottleneck Analysis

Based on this model, the system’s maximum theoretical throughput $\lambda_{\max}$ is constrained by the CPU-intensive stages rather than I/O. The condition for system stability is:

(4) $$\lambda_{\mathrm{in}}\leq \frac{1}{{\bar{t}}_{\mathrm{CPU}}}\approx \frac{1}{\Delta_{\mathrm{GIL}}\left(N\right)+t_{\mathrm{serialize}}}$$

This theoretical inequality explains the experimental phenomenon observed in Section 6.3, where CPU saturation (due to serialization and GIL overhead) becomes the bottleneck before network bandwidth is exhausted.

## 6. Performance Evaluation and Discussion

### 6.1. Experimental Setup

To quantify the performance of MIGS, a controlled testbed was established comprising:

- Gateway: Raspberry Pi 4 (Specifications as in Section 4.1).
- Network: Isolated Gigabit LAN to eliminate external traffic interference.
- Simulated Devices: A high-performance workstation running software simulators for 15 discrete devices (5x Modbus TCP servers, 5x OPC UA servers, 5x MQTT publishers).
- Cloud Endpoint: A local Mosquitto MQTT broker acting as the cloud receiver to measure latency without internet jitter.
- Metrics: End-to-end latency, system throughput (messages/second), CPU/Memory utilization, and packet loss rate.
- Metric Definitions: “Data rate” refers to the number of application-layer JSON payloads successfully transmitted to the Northbound broker per second. Memory usage was measured using the Python memory_profiler library and system-level ps command.

### 6.2. Latency Analysis

End-to-end latency is defined as the time delta between data generation at the sensor simulator and its successful reception at the cloud broker. Measurements were taken over a 1-h period for each scenario.As illustrated in Table 2.

The results indicate that latency scales linearly with device count. The 95th percentile latency of 108 ms for 15 devices is well within the acceptable range for most monitoring-based industrial applications, which typically require <200 ms response times [32]. The jitter observed at higher loads is primarily attributed to the OS scheduler context switching between the multiple Python threads.

### 6.3. Throughput and Scalability Analysis

Stress testing was conducted to determine the system’s breaking point. Table 3 summarizes the system throughput under various load conditions.

- 0–500 msgs/s: The system maintained 0% packet loss and stable CPU usage (∼40%).
- 1000 msgs/s: CPU utilization rose to ∼78%, but packet loss remained negligible (<0.2%).
- 1500 msgs/s: CPU utilization approached saturation (95.6%), and packet loss increased to 1.8%. This bottleneck is largely attributed to the Python Global Interpreter Lock (GIL) which limits true parallelism on multi-core CPUs, and the serialization overhead of JSON processing.
- Scalability Implication: While the current single-node implementation is sufficient for typical edge deployments (connecting 10–50 sensors), scaling to hundreds of devices would require either horizontal scaling (clustering multiple gateways) or migrating performance-critical components to a compiled language like Go or Rust.

### 6.4. Resource Utilization Profile

Long-term stability tests (24 h) showed:

- Memory: Stable consumption between 320–380 MB. The slight fluctuation correlates with the garbage collection cycles of the Python runtime.
- Cache Efficiency: Redis maintained a hit ratio of >99%, confirming that the decoupling strategy effectively isolates the Northbound publisher from Southbound acquisition latency.

### 6.5. Protocol-Specific Performance

Comparative analysis of different protocols revealed variations in performance characteristics (Table 4).The significantly higher connection time for OPC UA (245.8 ms) compared to Modbus (12.3 ms) is attributed to the complex handshake mechanism of the OPC UA binary protocol, which involves endpoint discovery, secure channel negotiation, and session activation. Modbus TCP, by contrast, requires only a standard TCP 3-way handshake.

Regarding resource consumption, memory usage per instance was quantified using the Python memory_profiler library, capturing the incremental RSS (Resident Set Size) growth upon spawning a new connector thread.

### 6.6. Comparative Discussion

Comparison with open-source Solutions:Unlike rigid open-source solutions (Table 5), MIGS offers superior deployment flexibility. The modular design allows developers to extend protocol support (e.g., adding BACnet) without recompiling the core kernel.

Table 5 presents a comparative analysis against two leading open-source edge platforms: Node-RED and EdgeX Foundry.

- vs. Node-RED: While Node-RED offers a lower baseline memory footprint (~150 MB), it operates as a single-threaded Node.js process. A blocking operation or unhandled exception in a single function node can potentially crash the entire runtime or stall other flows. MIGS addresses this by encapsulating each device driver in a protected thread, ensuring that a driver failure does not impact the core management services.
- vs. EdgeX Foundry: EdgeX provides superior process-level isolation through its microservices architecture. However, this comes at the cost of significant resource overhead (>800 MB RAM for a standard deployment) and deployment complexity (requiring orchestration of 10+ containers). MIGS strikes a balance, offering sufficient logical isolation for industrial device polling while maintaining a lightweight footprint suitable for resource-constrained gateways (e.g., Raspberry Pi 3/4) without the operational overhead of a full microservices stack.

### 6.7. Limitations

While MIGS demonstrates significant advantages in flexibility and protocol integration, several limitations inherent to its design and current implementation warrant discussion.

1. **Scalability Constraints under High Concurrency:** The current implementation leverages Python’s threading module for instance-based isolation. However, the Global Interpreter Lock (GIL) in CPython prevents true parallelism on multi-core processors, effectively serializing CPU-bound operations [33]. While our “one-thread-per-device” model works well for I/O-bound tasks (typical in IoT polling), performance degradation becomes observable when the number of concurrent devices exceeds 50 or when high-frequency data parsing (JSON serialization) saturates the CPU. Future iterations could mitigate this by adopting a multi-process architecture or migrating performance-critical components to a compiled language like Go or Rust.
2. **Single Point of Failure (SPoF):** As a centralized edge node, the gateway itself represents a single point of failure. Although the software architecture ensures that individual device driver crashes do not bring down the system (via process isolation), a hardware failure or OS-level crash would sever connectivity for all downstream devices [1]. The current prototype lacks a native High Availability (HA) mechanism, such as active-passive clustering or Virtual Router Redundancy Protocol (VRRP) support, which are essential for mission-critical industrial scenarios.
3. **Security Hardware Dependencies:** MIGS currently relies on software-based security mechanisms (TLS, Token Auth). It does not integrate with hardware-based security anchors like Trusted Platform Modules (TPM) or Hardware Security Modules (HSM) for secure key storage and boot integrity verification [34]. This leaves the system vulnerable to physical tampering or advanced persistent threats (APTs) where an attacker with root access could extract cryptographic keys.
4. **Deterministic Latency Limitations:** Running on a general-purpose operating system (Raspberry Pi OS/Debian) introduces non-deterministic latency due to OS scheduler preemption and background services [35]. Unlike Real-Time Operating Systems (RTOS), MIGS cannot guarantee hard real-time responses (e.g., <1 ms jitter) required for high-speed motion control applications [31]. Consequently, MIGS is best suited for monitoring, supervisory control, and soft real-time applications rather than closed-loop critical control.

## 7. Conclusions

This study presented MIGS, a modular, multi-protocol IoT gateway designed to mitigate interoperability challenges in heterogeneous environments. Through a decoupled architecture and instance-based task management, MIGS achieves robust connectivity across Modbus, OPC UA, and MQTT protocols. Experimental validation confirms its operational stability and low-latency performance suitable for industrial deployment.

The proposed system effectively addresses the trade-off between flexibility and performance. By leveraging a modular software design on commodity hardware, it lowers the barrier to entry for IIoT adoption.

**Figure 9:**
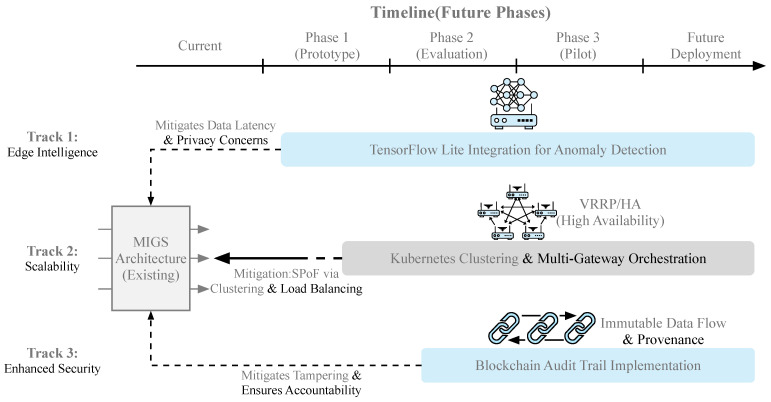
Future Work.

A systematic comparative analysis was conducted on gateway products offered by leading vendors in the market [36,37,38,39,40,41,42,43,44,45,46,47,48,49,50,51,52,53,54,55,56,57,58,59,60,61,62,63,64]. As illustrated in Figure 9, future work will focus on three key areas: (1) Edge Intelligence: Integrating lightweight TensorFlow Lite models for local anomaly detection; (2) Scalability: Implementing a Kubernetes-based orchestration layer for clustering multiple gateways; and (3) Enhanced Security: Integrating blockchain-based data integrity verification to create an immutable audit trail for sensor data.

## Figures and Tables

**Table 1:** Summary of key functional modules and their responsibilities.

Module	Key Responsibilities
Control Service	Provides Web API (Port 80) for user management and system configuration.
Connector Manager	Manages lifecycle (Init, Start, Stop) of all protocol connectors using Factory pattern.
Task Manager	Orchestrates atomic read/write tasks for each connection instance.
Cloud Platform Manager	Handles authentication and connection maintenance for external clouds (e.g., ThingsBoard).
OS Management	Monitors system health (CPU, RAM, Network) and handles network interface configuration.

**Table 2:** End-to-end latency measurements (ms).

Concurrent Devices	Min Latency	Max Latency	Avg. Latency	95th Percentile
5 devices	23	45	32	41
10 devices	28	78	45	69
15 devices	35	125	67	108

**Table 3:** System throughput under various conditions.

Data Rate (msgs/s)	CPU Utilization (%)	Memory Usage (MB)	Packet Loss (%)
100	18.5	245	0.0
500	42.3	318	0.0
1000	78.9	425	0.2
1500	95.6	587	1.8

**Table 4:** Protocol performance comparison.

Protocol	Connection Time (ms)	Data Point Transmission (ms)	Memory per Instance (KB)
Modbus TCP	12.3	8.7	156
OPC UA	245.8	15.2	892
MQTT	34.6	5.3	234

Note: The table demonstrates protocol performance metrics.

**Table 5:** Factual comparison with open-source baselines.

Feature	MIGS (Proposed)	Node-RED (v3.1)	EdgeX Foundry (v3.1)
Architecture	Modular Monolith (Python Threads)	Event-driven Flow (Node.js)	Microservices (Go)
Min. Memory Footprint	~350 MB (Measured)	~150 MB (Base)	>800 MB (Standard)
Fault Isolation	Logical (Thread Exception Handling)	None (Single Process Runtime)	High (Container/Process Level)
Configuration Update	Hot-reload (Zero Downtime)	Full Flow Redeploy (Brief Pause)	Dynamic via Consul Registry
Deployment Complexity	Low (Single Container/Script)	Low (Single Container/npm)	High (Docker Compose/K8s)

## Data Availability

The original contributions presented in this study are included in the article. Further inquiries can be directed to the corresponding author.

## Abbreviations

The following abbreviations are used in this manuscript:

IoT	Internet of Things
MIGS	Modular IoT Gateway System
MQTT	Message Queuing Telemetry Transport
OPC UA	OPC Unified Architecture
OS	Operating System
SSL	Secure Sockets Layer
IIoT	Industrial Internet of Things
GIL	Global Interpreter Lock
HMI	Human-Machine Interface
IPC	Inter-Process Communication
PAL	Protocol Abstraction Layer
LWT	Last Will and Testament
TLS	Transport Layer Security
RBAC	Role-Based Access Control
TPM	Trusted Platform Module
HSM	Hardware Security Module
QoS	Quality of Service
